# Huntingtin cleavage product A forms in neurons and is reduced by gamma-secretase inhibitors

**DOI:** 10.1186/1750-1326-5-58

**Published:** 2010-12-14

**Authors:** Kimberly B Kegel, Ellen Sapp, Jonathan Alexander, Patrick Reeves, Dorothee Bleckmann, Linsday Sobin, Nicholas Masso, Antonio Valencia, Hyunkyung Jeong, Dimitri Krainc, James Palacino, Daniel Curtis, Rainer Kuhn, Claudia Betschart, Miguel Sena-Esteves, Neil Aronin, Paolo Paganetti, Marian DiFiglia

**Affiliations:** 1 Department of Neurology, Massachusetts General Hospital, Charlestown, MA 02129, USA; 2 Nervous System Research, Novartis Institute for BioMedical Research, Novartis Pharma AG, Basel CH-4002, Switzerland; 3 Developmental & Molecular Pathways, Novartis Institute for BioMedical Research, Cambridge, MA 02139, USA; 4 Global Discovery Chemistry/Neuroscience, Novartis Institute for BioMedical Research, Novartis Pharma AG, Basel, CH-4002, Switzerland; 5 Department of Medicine and Cell Biology, University of Massachusetts Medical School, Worcester, MA 01655, USA

## Abstract

**Background:**

The mutation in Huntington's disease is a polyglutamine expansion near the N-terminus of huntingtin. Huntingtin expressed in immortalized neurons is cleaved near the N-terminus to form N-terminal polypeptides known as cleavage products A and B (cpA and cpB). CpA and cpB with polyglutamine expansion form inclusions in the nucleus and cytoplasm, respectively. The formation of cpA and cpB in primary neurons has not been established and the proteases involved in the formation of these fragments are unknown.

**Results:**

Delivery of htt cDNA into the mouse striatum using adeno-associated virus or into primary cortical neurons using lentivirus generated cpA and cpB, indicating that neurons in brain and *in vitro *can form these fragments. A screen of small molecule protease inhibitors introduced to clonal striatal X57 cells and HeLa cells identified compounds that reduced levels of cpA and are inhibitors of the aspartyl proteases cathepsin D and cathepsin E. The most effective compound, P1-N031, is a transition state mimetic for aspartyl proteases. By western blot analysis, cathepsin D was easily detected in clonal striatal X57 cells, mouse brain and primary neurons, whereas cathepsin E was only detectible in clonal striatal X57 cells. In primary neurons, levels of cleavage product A were not changed by the same compounds that were effective in clonal striatal cells or by mRNA silencing to partially reduce levels of cathepsin D. Instead, treating primary neurons with compounds that are known to inhibit gamma secretase activity either indirectly (Imatinib mesylate, Gleevec) or selectively (LY-411,575 or DAPT) reduced levels of cpA. LY-411,575 or DAPT also increased survival of primary neurons expressing endogenous full-length mutant huntingtin.

**Conclusion:**

We show that cpA and cpB are produced from a larger huntingtin fragment *in vivo *in mouse brain and in primary neuron cultures. The aspartyl protease involved in forming cpA has cathepsin-D like properties in immortalized neurons and gamma secretase-like properties in primary neurons, suggesting that cell type may be a critical factor that specifies the aspartyl protease responsible for cpA. Since gamma secretase inhibitors were also protective in primary neurons, further study of the role of gamma-secretase activity in HD neurons is justified.

## Background

Huntington disease (HD) is caused by an expansion of a normal CAG repeat in the gene encoding the protein huntingtin [1]. The CAG repeat is translated into a polyglutamine (Q) tract near the N-terminus of huntingtin, which is 3144 amino acids (aa) in length. Patients that bear this mutation suffer neurodegeneration resulting in cognitive and personality changes early in the disease, and later develop an overt movement disorder characterized by chorea, rigidity, and dysphagia. HD is eventually fatal. Brains of HD patients show atrophy of the cortex and profound cell loss in the striatum. The exact cause of neuronal dysfunction and cell death is not clear.

Compelling evidence points to a role for N-terminal huntingtin fragments with an expanded polyQ tract (mutant huntingtin) in HD pathogenesis. In human HD post-mortem tissue, cytoplasmic and nuclear inclusions were exclusively recognized by antibodies to epitopes within the N-terminus of huntingtin [2]. Similarly, N-terminal huntingtin fragments shorter than 342 aa were identified by epitope mapping in degenerating neurons in the brain of HD knock-in mice (HdhCAG150) which express endogenous mutant huntingtin [3]. These stable fragments of mutant huntingtin appear pre-symptomatically as early as 2 weeks postnatal, suggesting their formation precedes the onset of disease in mice [4]. Mutant N-terminal huntingtin fragments expressed in mouse brain and in cells form inclusions and cause toxicity [5-11]. Transgenic mice over-expressing short N-terminal huntingtin fragments including huntingtin aa1-89 [5] or huntingtin aa1-171 [12] develop a motor phenotype and some neuropathological features observed in human HD patients. Moreover, these two HD mouse models show earlier onset of symptoms and have more severe phenotypes than transgenic mice expressing larger fragments [13] or full-length mutant huntingtin [14-16]. The length of huntingtin fragment does not dictate toxicity, however, since shorter fragments are not consistently more toxic than larger fragments. For example, exogenous expression of mutant huntingtin aa1-208 showed more toxicity than huntingtin aa1-89 in transfected cells [3]. Similarly, in mice a transgene expressing mutant huntingtin aa1-119 called "short stop" generates inclusions without a motor phenotype [17] although animals expressing a longer fragment (aa1-171) do develop motor symptoms [12]. Thus, proteolysis of full-length huntingtin at particular sites may be necessary to produce toxicity.

Huntingtin is a substrate for multiple proteases and cleavage within its N-terminus could form specific toxic fragments. There are cleavage sites in huntingtin for different caspases (caspase 2,3 and 6), calpain, cathepsin D and thrombin within 600 residues from the N-terminus [18-21]. Studies suggest that unique protease products of mutant huntingtin are harmful *in vivo*. Caspase 6 cleavage in mutant huntingtin at amino acid 586 has been identified as a potential source of toxic mutant huntingtin fragments *in vivo*. HD transgenic mice (Yac128) expressing full-length mutant huntingtin with a point mutation at the 586 cleavage site are protected from motor deficits and neuropathology [22]. In contrast, the Yac128 HD transgenic mouse with a point mutation at 552, the caspase 2/3 cleavage site, still develops neuropathology and motor deficits [22]. The results suggest that the caspase-6 product (huntingtin aa1-586) is toxic whereas the caspase-2/3 product (huntingtin aa1-552) is not.

Smaller N-terminal cleavage products (cp) of mutant huntingtin known as cpA and cpB were first identified in cultured immortalized neurons over-expressing larger N-terminal huntingtin fragments or full-length mutant huntingtin [23]. Treating cells with proteasome inhibitors increased levels of the fragments. The accumulation of cpA and cpB in the presence of proteasome inhibitors may have relevance to the progressive and age dependent nature of HD, since proteasome activity decreases with normal aging [4]. The exact cleavage sites are unknown, but biochemical analysis and epitope mapping suggest that cpA is < 115 aa and cpB is < 214aa [23]. Deletion analyses support this conclusion: deletion of huntingtin aa104-114 impaired generation of cpA whereas deletion of huntingtin aa205-215 impaired formation of cpB [23,24]. Immunolabeling studies revealed that cpA can enter the nucleus and localize to intranuclear inclusions; CpB is excluded from the nucleus likely due to its larger size and instead is detected in cytoplasmic inclusions [23]. In mice, the addition of a nuclear localization signal to mutant htt 1-89 accelerates onset and progression of disease [25]; thus a proteolytic event in neurons that creates a huntingtin fragment in a similar size range such as cpA may hasten pathology in humans. In view of this, it is imperative to determine whether cpA and cpB occur in neurons and identify effective protease inhibitors that could have therapeutic benefits. It is not known if cpA or cpB can be detected in primary neurons and in the absence of proteasome inhibition. Moreover, the protease(s) involved in forming cpA and cpB have not been identified; however, some evidence indicates that an aspartyl protease, possibly cathepsin D, was responsible for cpA using a cell-free *in vitro *assay [23,24].

In this study we used viral delivery of truncated huntingtin into primary neurons and into brain to determine if neurons generate cpA and cpB. We also screened a focused library of protease inhibitors to identify the protease(s) involved in generating these fragments. Our findings suggest that neurons are capable of forming cpA and cpB *in vivo *and that an aspartyl protease activity generates cpA.

## Results

### CpA and cpB form in neurons *in vivo *and *in vitro*

**Figure 1:**
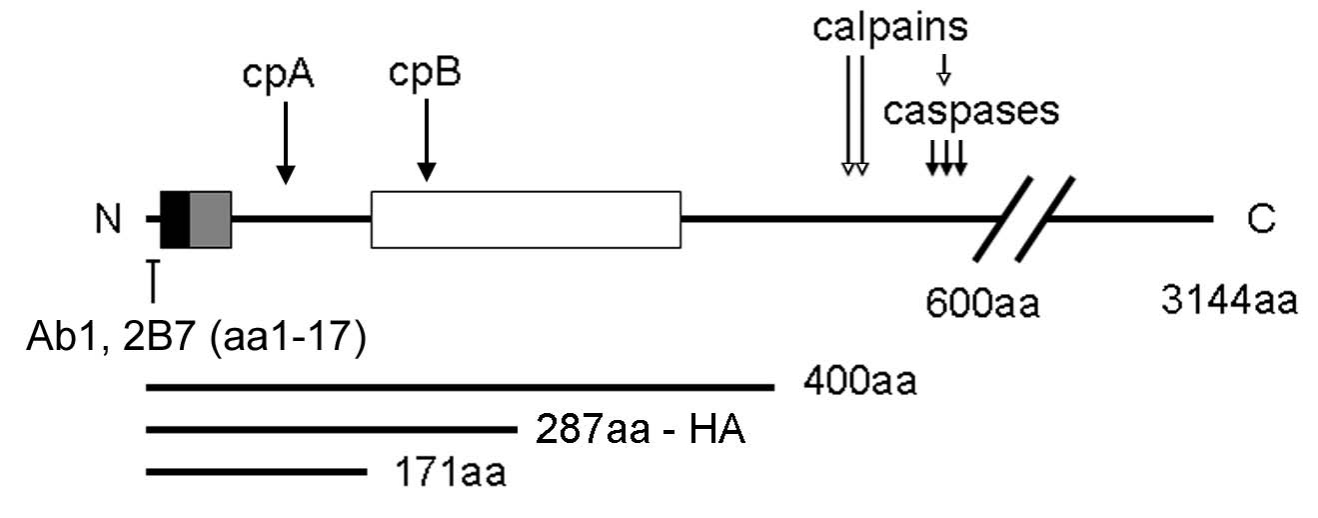
**Schematic of huntingtin protein**. Schematic shows locations of epitope for Ab1 antibody (aa1-17) and mAb 2B7 (aa1-17) and the polyglutamine region (black box). Membrane binding is contributed by aa1-17 [44], the poly-proline region (gray box, [45]) and aa172-372 (white box, [62]). Regions of calpain and caspase cleavage are represented by arrows. Approximate regions of cpA and cpB cleavage are also marked by arrows. Shown below are three huntingtin cDNA constructs used in this study.

**Figure 2:**
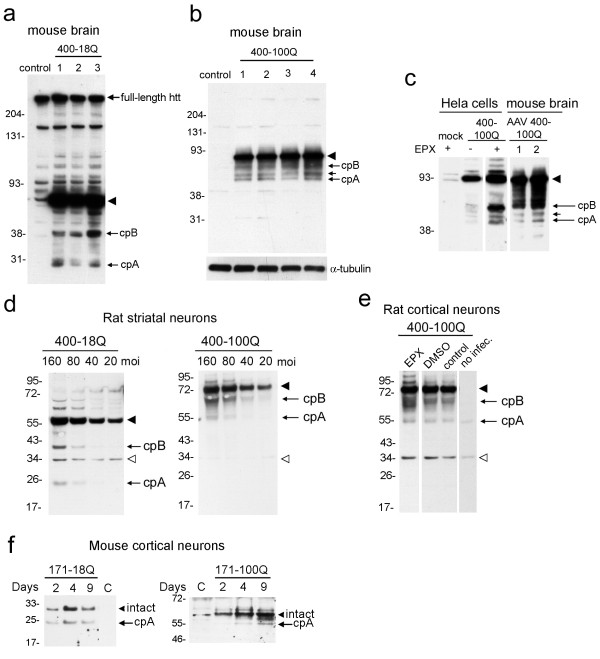
**Primary neurons in culture and in brain produce and accumulate cpA from over-expressed htt1-400**. **(a, b) **Western blot shows lysates from striatum of 3 mice injected with AAV-htt1-400-18Q and 4 mice injected with AAV-htt1-400-100Q probed with anti-huntingtin antibody Ab1. Arrowheads show the expressed, intact htt1-400-18Q (a) and htt1-400-100Q (b), and arrows indicate cpA and cpB. Short unlabeled arrow indicates an intermediate band (b). For (a) 4-12% Bis-Tris gel and full-length huntingtin serves as a loading control, and for (b) 4-20% Tris-glycine gel and blot was reprobed with tubulin as a loading control. **(c) **Lysates from infected mouse brain striatum were also analyzed on the same gel as lysates from HeLa cells expressing htt400-100Q ± the proteasome inhibitor epoxomicin (EPX) to identify cpA and cpB from mouse brain. SDS-PAGE run on 10% Tris-glycine gel. **(d) **Western blots of lysates from primary rat striatal neurons treated with lentivirus delivering cDNAs encoding htt1-400-18Q or htt1-400-100Q. Concentration of lentivirus in multiplicity of infection (moi) indicated at top. Arrowhead, intact htt1-400; arrows, cpA and cpB, open arrowhead, non-specific band. 4-12% Bis-Tris gels. **(e) **Western blots of lysates from primary rat cortical neurons treated with lentivirus (80 moi) delivering cDNAs encoding htt1-400-100Q. Arrowhead, intact htt1-400; arrows, cpA and cpB; open arrowhead, non-specific band. 3-8% Tris-acetate gel. Cells were treated with 5 M epoxomicin (EPX) or carrier (DMSO) for final 4 hours. **(f) **Western blots of lysates from primary cortical mouse neurons infected with lentivirus-PGK-htt1-171 (18Q or 100Q) then harvested post-infection at days indicated. Lysates from uninfected control "C" neurons are shown for comparison. Arrowheads, intact expressed protein; arrows indicate cpA. For 171-100Q, an intermediate band is also visible. 4-12% Bis-Tris gel for 18Q and 3-8% Tris-acetate gel for 100Q. Samples were run on the same gel, white line indicates removal of lanes.

Small molecular weight cleavage products of huntingtin in the range of cpA or cpB have been identified in brain lysates from human control and HD brain [6,26], in transgenic mice over-expressing huntingtin aa1-171 [2] and in HD knock-in mice expressing Q150/Q150 [24]. It is unclear if the fragments detected in brain lysates correspond to cpA and cpB and if they form in neurons or in glia, the non-neuronal cells of the brain. To address this directly, we injected mouse striatum with adeno-associated virus (AAV) vector encoding cDNA for truncated wild-type huntingtin (htt1-400-18Q) or mutant huntingtin (htt1-400-100Q) (Figure 1). We used cDNA encoding truncated huntingtin because viral vectors cannot accommodate encoded full-length huntingtin cDNA ( > 9 kb); also levels of N-terminal huntingtin fragments expressed from the endogenous full-length huntingtin are difficult to detect even in the presence of proteasome inhibitors. Recent studies showed that huntingtin aa1-400 delivered by AAV is expressed in striatal and cortical neurons and not in non-neuronal cells and causes neuropathology and a motor deficit [11]. Biochemical analysis of striatal lysates from brain infected with AAV expressing htt1-400-18Q and htt1-400-100Q revealed proteolytic products of the expected size for cpA (25 kDa for wild-type and 50-55 kDa for mutant) and cpB (35-40 kDa from wild-type and 60 kDa from mutant) (Figure 2a, b). Note that polyQ expansion slows the migration of intact and cleaved huntingtin on SDS-PAGE. An intermediate band was also detected with htt-400-100Q as previously described [24]. The products were absent in control (uninfected) brains. To confirm the products in brain were cpA and cpB, we compared the migration of the fragments from the brain lysates with lysates from HeLa cells expressing htt1-400-100Q treated without or with the proteasome inhibitor epoxomicin (5 μM), which increased levels of cp A and cpB in the cultured cells (Figure 2c). In AAV-infected mouse brains expressing htt1-400-100Q, proteolytic products migrated similarly to cpA and cpB in HeLa cells. These data show that cpA and cpB accumulate in neurons in a mouse model of HD.

To establish that cpA and cpB also formed in neurons *in vitro*, we infected rat primary striatal neurons with different concentrations of lentivirus encoding htt1-400-18Q or 1-400-100Q. We used rat embryos instead of mouse embryos since the striata of rat embryos is technically easier to dissect and yields more neurons. After two weeks in culture, huntingtin fragments migrating at the size of cpA and cpB were detected by western blot in lysates of the infected primary striatal neurons; the levels of the fragments increased with increasing concentration of virus (Figure 2d). We also observed cpA and cpB in cortical rat neurons infected with lentivirus htt1-400-100Q after two weeks in culture, and the fragments were enhanced by treatment with 5 μM epoxomicin applied 4 hours prior to harvest (Figure 2e). With epoxomicin, levels of cpB increased 52.4% compared to DMSO alone and levels of cpA increased 37.6% compared to DMSO alone (P < 0.05, n = 3, unpaired t test). Based on deletion analysis, huntingtin truncation to amino acid 171 should result in formation of cpA but not cpB [23,24]. We infected primary mouse cortical neurons with lentivirus containing cDNAs encoding huntingtin aa1-171 (18Q and 100Q) driven by a PGK promoter. Previous study has shown that use of PGK promoter is more effective than CMV promoter for lentiviral infection of mutant huntingtin in neurons [27]. In primary mouse cortical neurons infected with lenti-PGK-htt1-171 (18Q and 100Q), the intact expressed protein and the protease product cpA were detected from 2-9 days post-infection in the absence of epoxomicin (Figure 2f). Levels of htt171-18Q and htt171-100Q accumulated from 2 to 4 days post infection. However at 9 days post-infection, levels of intact htt171-100Q continued to increase whereas levels of htt171-18Q had peaked and decreased (Figure 2f). CpA continually formed from htt171-18Q but was produced more slowly from htt171-100Q. CpA also formed in primary rat neurons infected with Lenti-PGK-htt1-171 (18Q & 100Q) and was detectible in the absence of epoxomicin (not shown). These studies suggest that cpA and cpB form in neurons in brain and *in vitro*. Detection of cpA and cpB production in neurons in brain and *in vitro *did not require addition of proteasome inhibitors. Moreover, mutant htt171-100Q may resist cleavage and degradation.

### Cell-based screen for inhibitors of wild-type huntingtin cpA and cpB

**Figure 3:**
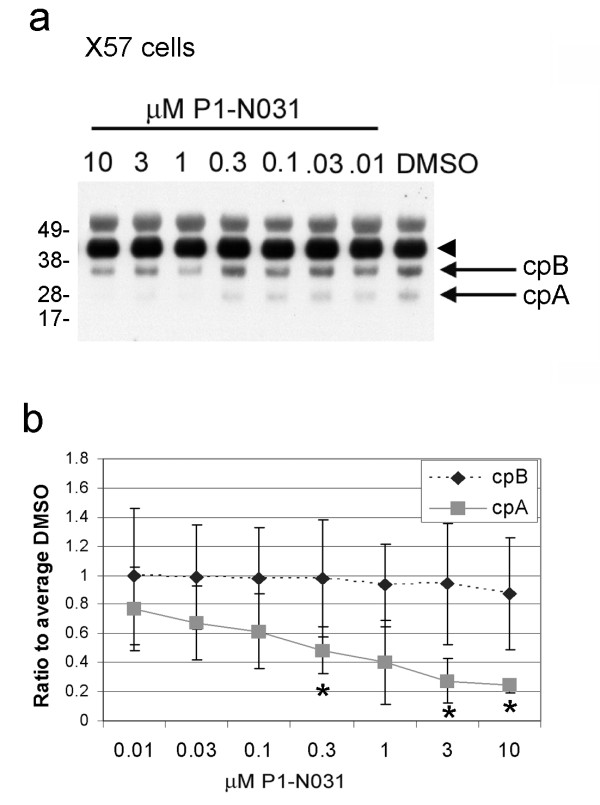
**Compound P1-N031 shows dose-dependent inhibition of cpA in X57 cells**. **(a) **Example western blot of lysates from clonal striatal cells transfected with H287-18Q-HA and treated with small molecule P1-N031 (Novartis) or carrier (DMSO). Arrowhead identifies the intact htt1-287-HA and arrows show cpA and cpB. The compound P1-N031 inhibits in a dose-dependent manner levels of cpA, but not cpB. **(b) **Dose-response curves for cpA (solid line) and cpB (dashed line). Y-axis is densitometry of ratio of signal for protease fragment relative to intact Htt1-287 to average DMSO. X-axis is concentration of compound in μM. Error bars represent sd, * p < 0.05, Friedman's test with Dunn's multiple comparison post-test (N = 3 /dose).

**Figure 4:**
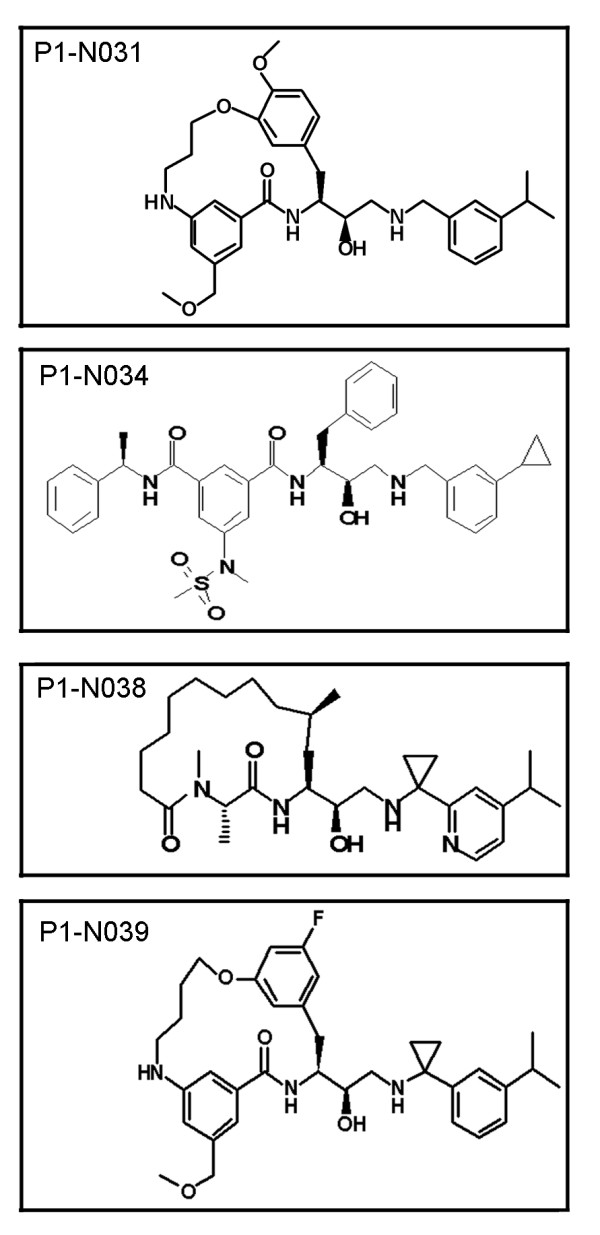
**The chemical structures for select aspartyl proteases**. Compounds P1-N031, P1-N034 and P1-N039 were effective at inhibiting cpA in clonal striatal cells. Compound P1-N031 has greater specificity for cathepsin D and cathepsin E compared to BACE 1 and BACE 2 whereas compound P1-N038 which was ineffective at blocking cpA has greater specificity for BACE 1 and BACE 2 (see also Table 1).

The aspartyl protease inhibitor pepstatin A was found effective in partially reducing levels of cpA *in vitro *in cell-free digestion assays or in immortalized neuronal-like cells when used at 100 μM but had no effect on cpB [23,24]. Pepstatin A inhibits a broad spectrum of aspartyl proteases and is poorly cell permeable and therefore not a reliable inhibitor for cell based studies. Since the protease for cpB was also unknown, we performed a cell-based assay to find soluble compounds that inhibit cpA and/or cpB production (Additional File 1** Figure S1**). Compounds with inhibitory properties against specific aspartyl proteases were included in the screen. Clonal striatal (X57) cells are immortalized cells formed from a fusion of mouse neuroblastoma and mouse embryonic striatum [28] and were used for the screen due to the large number of compounds. We transiently transfected X57 cells with htt1-287-18Q with a C-terminal HA tag (H287-18Q-HA) and treated with epoxomicin for the final 6 hours to increase the levels of cpA and cpB (Figure 3a) as described by [24]. We screened a focused library of 102 proprietary compounds known to inhibit different families of proteases. Compounds were screened at 10 μM. 17 hits representing 5 major categories of proteases were identified that inhibited cpA and/or cpB production by > 50% (Additional File 2** Table S1**). Of the 17 primary hits, only three compounds showed dose-responsive inhibition of cpA (one example illustrated in Figure 3). All three were inhibitors of aspartyl proteases (Table 1** and **Figure 4). The most effective compound in cells, P1-N031, shows the highest specificity for cathepsin E and greater specificity for human cathepsin D compared to BACE1, BACE2, or (porcine) pepsin measured using *in vitro *enzyme assays (Table 1). A gamma secretase inhibitor was also included and was not effective in the screen with clonal striatal cells. We also expressed htt1-287-18Q-HA in a human cell line (HeLa) and detected cpA. Aspartyl protease inhibitors were effective at inhibiting cpA formed in HeLa cells. However, the inhibitors that reduced levels of cpA in HeLa cells differed from those identified using X57 cells. Compound P1-N039, which is highly effective at blocking both cathepsin D and cathepsin E, reduced cpA in HeLa cells, whereas compounds P1-N031 and P1-N034 were not effective in HeLa cells (data not shown). The other inhibitors effective in HeLa cells were specific for cathepsin D over cathepsin E. No hits were identified that inhibited cpB production in a dose-responsive manner.

**Table 1:** Selectivity of inhibitors against cpA production in clonal striatal X57 cells (EC50) compared to selectivity against purified aspartic proteases (IC50) in μM.

Compound	Human Htt cpA EC_50_	Human BACE1 IC_50_	Human BACE2 IC_50_	Human Cathepsin D IC_50_	Human Cathepsin E IC_50_	Porcine Pepsin IC_50_
**P1-N031**	0.2	5.5	3.1	0.018	0.0002	0.29
**P1-N034**	4.9	< 0.0005	0.0006	~0.5	0.002	0.79
**P1-N038**	> 10	0.007	0.014	0.52	0.81	0.14
**P1-N039**	1.3	0.003	0.003	0.0006	0.0002	0.0002

### Cell-free screen to detect inhibitors of huntingtin cpA and cpB

Previous studies showed that in a cell-free assay of huntingtin cleavage, pepstatin A blocked formation of cpA [23,24]. In this assay, the crude nuclear pellet containing lysosomes, ER and other membranes, as well as expressed huntingtin protein is isolated from cells and after adjusting the pH to 3.5, allowed to self-digest for 1 hour at 37°C. We performed a cell-free assay using lysates from X57 or HeLa cells expressing H287-18Q-HA. Two compounds that inhibited cpA in X57 cells in the cell-based screen (P1-N031 and P1-N039) were effective in reducing levels of cpA in the cell-free assay (Additional File 1** Figure S2a, b**). The same compounds inhibited cpA-like fragments in digested HeLa cell lysates expressing H287-18Q-HA or mutant htt1-400-100Q (**compare **Additional File 1** Figure S2b and c**).

Both P1-N031 and P1N039 preferentially inhibit cathepsin D and cathepsin E in purified *in vitro *enzyme assays. Compound P1-N038, which has a greater specificity for BACE1 and BACE2 (Table 1), failed to inhibit cpA formation in the self-digestion assay and in the cell-based assay. The findings in the cell-free assay support the results in the cell-based assay and suggest that the aspartyl protease responsible for creating cpA from huntingtin in clonal striatal cells is related to cathepsin D or cathepsin E.

### Effects of selected aspartyl protease inhibitors on cpA production in primary neurons and evaluation of cathepsin D as the responsible protease

We tested if the aspartyl protease inhibitors that inhibited cpA in X57 cells were also effective in reducing cpA production in rodent primary neurons. We used lenti-htt1-171-18Q, which produces cpA (Figure 2f), to test compounds in primary rat and mouse neurons. We examined in primary neurons the three compounds that were effective in clonal striatal X57cells (P1-N031, P1-N034, P1-N039). Primary cortical neurons from rat exposed to several of the compounds at 3-10 μM showed morphological features of cell death including blebbing, cell body shrinkage and detachment from the dish. This occurred in the absence of infection with lentivirus-htt1-171. Therefore, we treated cells with 1 μM, which did not produce these toxic changes. Compounds were introduced into rat neurons 18 hours after infection with lentivirus-htt1-171-18Q. We also evaluated the effects of the compounds in mouse neurons with the same protocol and by pre-treating with compounds 18 hours before infection with lentivirus. None of the compounds were effective at inhibiting cpA in neurons at 1 μM in either of the experimental paradigms tested (data not shown). We also treated neurons with 100 μM pepstatin A or a peptide inhibitor of cathepsin D (10 μM H-GEG), both of which prevent accumulation of cpA in clonal striatal X57 cells [24]. These two inhibitors were not toxic but were ineffective in inhibiting cpA in neurons. These results show that none of the aspartyl protease inhibitors with high specificity for cathepsin D were effective in primary neurons.

Western blot analysis confirmed that cathepsin D is expressed in clonal striatal X57 cells, in brain and in primary neurons whereas cathepsin E was only detectible in X57 cells and at very low levels in primary neurons (Additional File 1** Figure S3**). We introduced a siRNA targeting cathepsin D to reduce levels of cathepsin D protein in mouse primary neurons; mRNA silencing partially reduced protein levels of cathepsin D but had no effect on levels of cpA (Additional File 1** Figure S4**). Together with the results above using cathepsin D inhibitors, these findings suggest that cathepsin D is not the aspartyl protease responsible for cleavage of huntingtin to generate cpA in primary neurons.

### Modulators and inhibitors of gamma-secretase reduce levels of cpA in primary neurons

**Figure 5:**
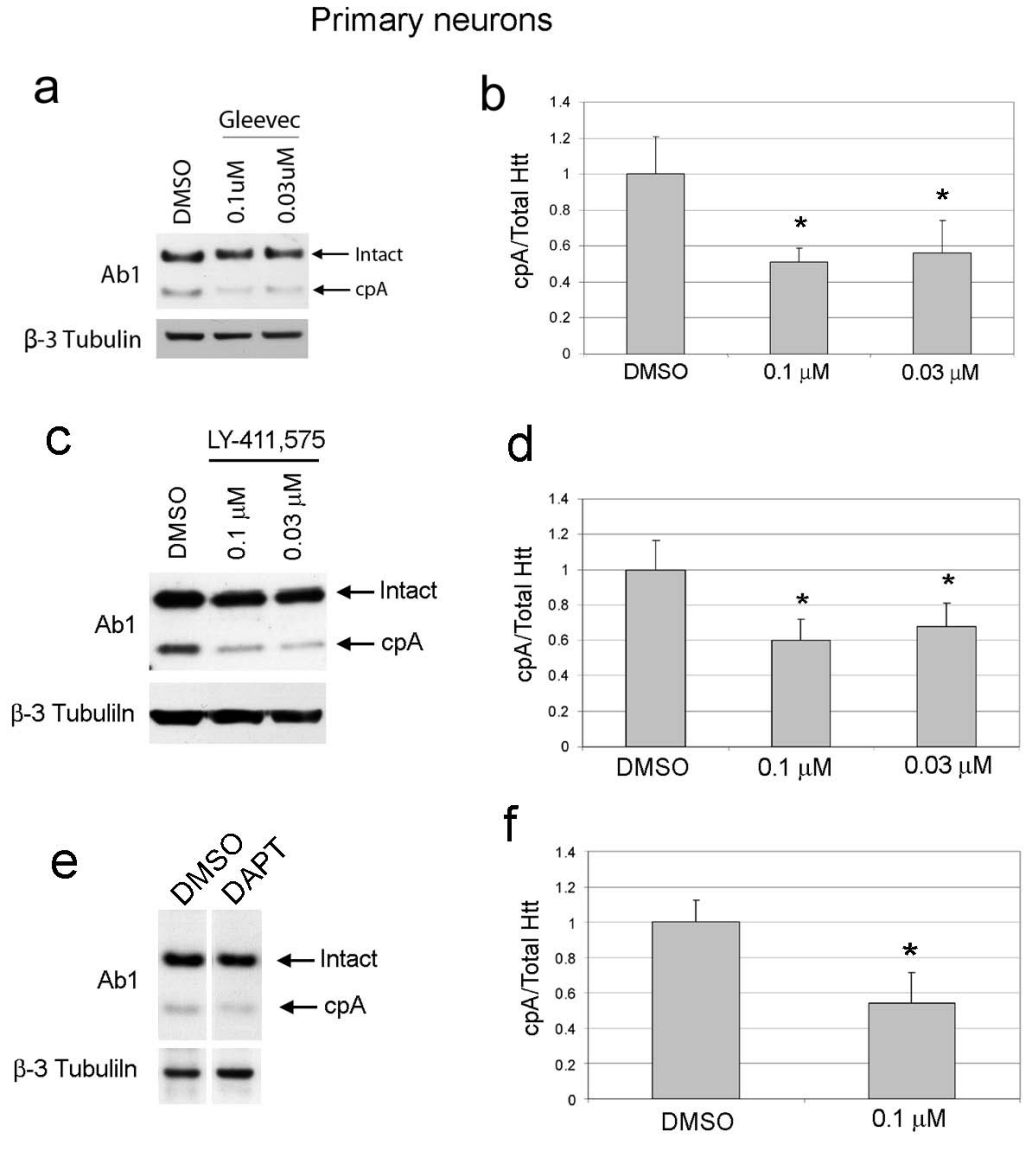
**Modulators and inhibitors of gamma secretase reduce CpA levels produced from htt171-18Q in primary mouse neurons**. (**a**) Western blot of lysates from neurons treated with Gleevec for 18 hours then infected with lentiPGK-ht171-18Q and cultured an additional 24 hours. Arrows indicates the over-expressed huntingtin fragment (Intact) and the cleavage product, cpA. Blots were re-probed with beta-3-tubulin to control for total neuron protein. (**b**) Densitometry results from western blot analysis of Gleevec treatment. Levels of cpA were significantly reduced in cultures treated with indicated concentrations compared treatment with DMSO solvent alone. One way ANOVA, p < 0.03, * p < 0.05 Dunnett's Multiple comparison test, n = 3 for DMSO and n = 3 for treatment conditions. (**c**) Western blot of lysates from neurons infected with lentiPGK-ht171-18Q for 18 hours then treated with LY-411,575 and cultured an additional 24 hours. Arrows indicates the over-expressed huntingtin fragment (Intact) and the cleavage product, cpA. Blots were re-probed with beta-3-tubulin to control for total neuron protein. (**d**) Densitometry results from western blot analysis of LY-411,575 treatment. Levels of cpA were significantly reduced in cultures treated with indicated concentrations compared treatment with DMSO solvent alone. One way ANOVA, p < 0.003, * p < 0.05 Dunnett's Multiple comparison test, n = 3 for DMSO and n = 3 for treatment conditions. (**e**) Western blot of lysates from neurons infected with lentiPGK-ht171-18Q for 18 hours then treated with DAPT at 0.01 μM and cultured an additional 24 hours. Arrows indicates the over-expressed huntingtin fragment (Intact) and the cleavage product, cpA. Blots were re-probed with beta-3-tubulin to control for total neuron protein. (**f**) Densitometry results from western blot analysis of DAPT treatment. Levels of cpA were significantly reduced in cultures treated with indicated concentrations compared treatment with DMSO solvent alone. * p < 0.05, n = 3 for DMSO and n = 3 for treatment conditions, paired t-test.

We found a different set of aspartyl protease inhibitors was effective in clonal striatal cells versus HeLa cells. Thus, we speculated that the protease cleaving cpA may differ among cell types. Despite the fact that an inhibitor of the aspartyl protease, gamma secretase, was among the compounds that failed to affect cpA in clonal striatal cells, we thought gamma secretase could be a candidate for generating cpA in neurons. Gamma-secretase is expressed in neurons and is associated with membranes where huntingtin is also localized [29]. With this in mind, we tested compounds known to inhibit gamma-secretase in primary neurons. The kinase inhibitor imatinib mesylate (Gleevec) retards production of beta-amyloid from amyloid precursor protein through effects on gamma-secretase [30,31]. We tested whether cpA levels were affected by Gleevec. Primary neurons were infected with lenti-htt1-171-18Q and treated with Gleevec at 0.1 μM and 0.03 μM. Gleevec significantly reduced levels of cpA compared to treatment with DMSO carrier alone (Figure 5a, b). These findings show that the protease responsible for cleaving huntingtin to produce cpA can be modulated by Gleevec. We also tested two specific inhibitors of gamma secretase that are mechanistically distinct: LY-411575 [32] and DAPT [33]. We found that LY-411575 inhibited cpA production in primary neurons (Figure 5c, d). Treatment with 0.1 μM DAPT was also effective at inhibiting cpA production in primary neurons (Figure 5e,f). These results suggest that gamma-secretase is responsible for cpA production in primary cortical neurons.

We examined the effects of a cell free source of gamma-secretase activity on huntingtin proteolysis. SY5Y cells are reported to contain functionally active gamma-secretase complex [34]. After 2 or 5 hours incubation with a CHAPSO-soluble membrane fraction of SY5Y cells prepared according to Li et al. [35], there was no proteolysis of immunopurified FLAG-huntingtin 171-18Q (Fhtt171-18Q) observed (results not shown).

### Gamma-secretase inhibitors increase survival of HD primary neurons

**Figure 6:**
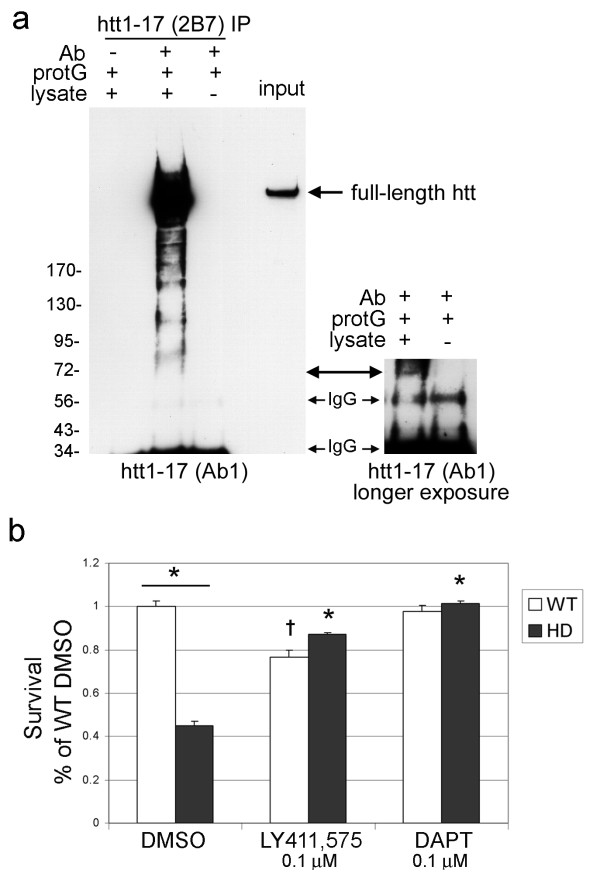
**Inhibitors of gamma-secretase protect primary cortical HD neurons homozygous for full-length mutant huntingtin (Q140/Q140) from cell death**. (a) Immunoprecipitation (IP) of mutant huntingtin from 23 DIV neurons using a monoclonal antibody against htt1-17 (mAb2B7) and probed with polyclonal anti-htt1-17 (Ab1). Arrow indicates full-length huntingtin when IP is performed with mAb2B7 while the control lanes are blank. N-terminal fragments were detected at ~100 kDa and ~80 kDa. Small blot on right is the enlargement of the second and third IP lanes with the double-sided arrow indicating a fragment ~72 kDa. The predicted mobility of cpA with 140Q ranges from ~69 kDa to ~85 kDa. (**b**) Graph shows MTT conversion reported as mean percentage of wild-type (WT) neurons treated with carrier (DMSO) ±SD. One-way ANOVA, p < 0.001, Bonferroni's Multiple comparison tests: * over bar indicates p < 0.001 for WT vs HD, *p < 0.001 compared to HD treated with DMSO, ^†^p < 0.001 compared to WT treated with DMSO.

Previously we showed reduced survival of HD primary neurons homozygous for a knock-in of 140 CAGs (Q140/Q140) at 10 DIV [36,37]. We questioned whether gamma secretase inhibition could reduce mutant huntingtin-dependent toxicity. First we used immunoprecipitation assay to look for evidence of small N-terminal fragments in Q140/Q140 primary cortical neurons since this method enhances detection of small N-terminal fragments generated from full-length huntingtin [24,38]. Following immunoprecipitation of huntingtin with monoclonal antibody (mAb) 2B7 against huntingtin 1-17 [39], fragments in the size range expected for cpA and cpB bearing a 140Q repeat were detected on western blots probed with polyclonal Ab1 against huntingtin 1-17 (Figure 6a). Having established the presence of small huntingtin fragments in HD primary neurons, we treated wild-type (WT) or HD cortical neurons with gamma-secretase inhibitors at 4DIV using a concentration (0.1 μM) that had previously reduced levels of cpA about 50%. Neuronal viability was measured by MTT assay at DIV 10. Both DAPT and LY-411,575 conferred significant protection to HD neurons (Figure 6b). LY-411,575 was slightly toxic to WT neurons under the prolonged treatment conditions (from 4DIV to 10DIV). Long-term treatment of WT primary neurons with Gleevec resulted in neurite retraction, possibly due to blockade of PI 3-kinase dependent signaling pathways, and thus was not evaluated in HD neurons. To ascertain if the increased survival in the presence of gamma secretase inhibitors correlated with reduced proteolysis of huntingtin, we treated neurons with DAPT or DMSO from 4 DIV to 10 DIV then immunoprecipitated full-length huntingtin and fragments using mAb 2B7. Western blots of immunoprecipitates probed with Ab1 showed a lower level of two mutant huntingtin fragments: one in the size range of cpA with a 140Q (~ 80 kDa) and a higher band at ~100 kDa (Additional File 1** Figure S5**). When standardized to the amount of full-length mutant huntingtin pulled down, the fragments from neurons treated with DAPT were at levels of 30% and 41% of DMSO for the 100 kDa band and 80 kDa band, respectively using densitometry. These results show that small fragments are produced in primary neurons expressing full-length endogenous mutant huntingtin and reduced levels of huntingtin fragments may mediate protective effects of gamma-secretase inhibitors.

## Discussion

Small N-terminal fragments of mutant huntingtin may be pathogenic in HD. In this study we sought to establish that huntingtin fragments cpA and cpB can form in neurons and to identify the protease(s) involved in formation of cpA and cpB in neurons. We found that cpA and cpB are detected in neurons and in mouse brain, under conditions where truncated wild-type or mutant huntingtin are over-expressed. Levels of cpA formed from truncated huntingtin in immortalized clonal striatal cells were diminished by inhibitors of aspartyl proteases selective for cathepsin D and cathepsin E. In primary neurons, cpA production was inhibited by Gleevec, a known modulator of beta-amyloid production and by the specific gamma-secretase inhibitors LY-411,575 and DAPT. Gamma-secretase inhibitors also increased survival of HD neurons. These findings suggest that the source of aspartyl protease activity required for cpA production may be cell specific and that gamma secretase activity may have a pathogenic role in HD through formation of cpA.

In the present study as in previous studies the addition of a proteasome inhibitor to immortalized cells expressing truncated huntingtin greatly enhanced detection of cpA and cpB. This suggests that these fragments are normally at low levels and are cleared very rapidly by the proteasome [23,24]. We were able to detect cpA and cpB in primary neurons and in the brain without the addition of proteasome inhibitors using expression of truncated huntingtin. These data suggest that the levels of cpA and cpB are more stable in neurons *in vitro *than in immortalized cells. Proteasome activity is higher in glia than in neurons [40]. It is possible that basal proteasome activity in immortalized neuronal cells is greater than in neurons because they are a fusion of neurons and glia. Previous investigators have speculated that N-terminal huntingtin fragments similar in size to cpA and cpB may accumulate in neurons due to a decline in proteasome function that occurs with normal aging [4].

Our study is the first to use a focused library of small compounds in a cell-based screen to identify a protease involved in huntingtin processing. The compounds contained in this library include inhibitors for several families of proteases namely cysteine proteases (calpain, caspases, cathepsins), serine proteases (thrombin/trypsin/chymotrypsin, kallikrein, and DPP), metalloproteases, aspartyl proteases, and the proteasome. Inhibitors spanning five protease families reduced cpB or cpA at 10 μM in the primary screen; however, only a small number of the aspartyl protease inhibitors attenuated cpA production in subsequent dose-dependent analysis in our cell-based assay. Our results support the conclusion that cpA is generated directly by an aspartyl protease. Indirect or non-specific effects on huntingtin cleavage may have accounted for the inhibition of cpA and cpB by non-aspartyl protease inhibitors in the initial cell based screen. The small compound hits proved toxic in neurons at comparable dilutions to those used in clonal striatal cells (X57 cells). Immortalized cells might be protected because they may express the multi-drug resistance pump, which can pump toxic moieties out of the cell. We used a self-digestion assay to verify the results of our cell-based screen. The huntingtin fragments in the self-digestion assay varied slightly in size compared to cpA accumulating in cells [4,23,24]. We suggest that these variations in size of the fragments between the two assays are most likely due to post-translational modifications such as ubiquitination [23] and not due to a difference in the protease since the same compounds were effective in both the cell-free and the cell-based assays.

Compound P1-N031 was the most effective in blocking cpA production in immortalized clonal striatal cells; this compound is an aspartyl protease transition state mimetic and has high specificity for cathepsin D and cathepsin E using *in vitro *enzyme assays. We conclude that in immortalized clonal striatal cells (X57 cells) huntingtin is subject to proteolysis by an aspartyl protease with properties similar to cathepsin D or cathepsin E. We cannot rule out a role for both cathepsin D and cathepsin E in cpA production in X57 cells, since these cells were enriched in both proteases.

To our surprise we found that three inhibitors of gamma-secretase activity reduced cpA levels in primary neurons. Gamma-secretase is a multiprotein complex expressed in neurons with aspartyl protease activity that localizes to the plasma membrane [41]. All known gamma-secretase substrates are type I integral membrane proteins [42]. Huntingtin does not contain a predicted transmembrane domain but does associates with membranes including plasma membranes through electrostatic and hydrophobic interactions via a membrane association domain at aa172-373 [43]. Regions within huntingtin aa1-89 (exon1) can also bind and insert into synthetic lipid bilayers [43-45] and increased polyglutamine length may increase membrane association and bilayer insertion [46]. Polyglutamine stretches can form pores in lipid bilayers [47], thus the polyglutamine region might mimic a transmembrane domain. Proteolysis by gamma-secretase generally occurs after removal of an ectodomain in the substrate by another protease or "sheddase" [48]. Huntingtin is cleaved at several sites distal to the cpA site: from aa453-586 by caspases, at ~aa400 by calpains and a metalloprotease, and by an unknown protease to create cpB (see Figure 1). Proteolysis at any of these sites might be analogous to ectodomain "shedding" leaving a short piece of huntingtin tightly associated with, and perhaps partially inserted into, the membrane. This may trigger a signal for proteolytic degradation by gamma-secretase [48]. It is also possible that huntingtin may be exposed to the catalytic pocket of gamma secretase at the aqueous surface of the membrane where protease activity is also speculated to occur [49]. We observed that mutant huntingtin resists cleavage consistent with another report [50]. Decreased flexibility of the polyglutamine and polyproline stretch in mutant huntingtin could impede entry of the peptide into the active site. The signal peptide peptidases, a related family of peptidases to gamma secretase, are intramembrane peptidases that are sensitive to compounds that affect gamma-secretase activity and could be responsible for cpA production. However, DAPT, which inhibited cpA production, does not affect their activity [51]. Recently an unbiased screen of siRNAs that target proteases identified eleven proteases that when knocked down reduced the production of a small huntingtin N-terminal fragment through cleavage at a more distal site [21]; one of these proteases, IMP5/signal peptide peptidase-like candidate protease 2C (SPPL2c) is related to gamma-secretase and might also produce cpA. It is possible that cpA is generated by another protease whose activation requires gamma-secretase activity. Gamma-secretase may liberate a C-terminal domain from a protein such as APP or Notch that induces transcription of the responsible protease, or gamma-secretase may directly activate another protease by removing its prodomain. A gamma-secretase cleavage assay failed to cleave htt1-171 -18Q. However, gamma-secretase activity can be modulated by substrate-specific activating factors [52] that may not be present in SY5Y cells and by specific phospholipids [53] which were not added to our reaction; one or more of these factors may be required for optimal activity against huntingtin. Thus, further studies will be required to discern direct or indirect roles of gamma-secretase activity in htt cleavage and minimal conditions for proteolysis.

We found two inhibitors of gamma-secretase activity are neuroprotective in HD140Q/140Q neurons. Our data also show that neuroprotection by one of these inhibitors (DAPT) was associated with reduced levels of two mutant huntingtin fragments one of which was consistent with the size of cpA. These findings implicate inhibition of a toxic fragment in neuroprotection. However, gamma-secretase inhibitors affect other signaling pathways such as calcium signaling [54]; thus, neuroprotection provided by gamma secretase inhibitors could also involve mechanisms unrelated to mutant htt processing.

Prior to its identification, gamma-secretase was described as an aspartyl protease with cathepsin D-like properties based on the ability of some peptidomimetic probes to inhibit both gamma-secretase activity and cathepsin D [55]. We found active cathepsin D in primary cortical neurons. However, compounds known to inhibit cathepsin D and genetic knockdown of the protease using siRNA failed to affect levels of cpA, supporting a conclusion that cathepsin D is not the responsible enzyme for cpA in neurons. One caveat to this conclusion is that we could only achieve 38% reduction in the levels of cathepsin D compared to the non-target siRNA control condition. Cathepsin D normally may be at saturating levels such that partial knockdown (38%) still shows no effect on cpA. One would still expect cathepsin D inhibitors to block cpA formation which we did not observe. In contrast to cathepsin D, we did not detect cathepsin E in 8-10 week old mouse brain lysates or in primary neurons. Previous studies showed that cathepsin E is undetectable in rat brain from young animals (2 months) or found only in microglia of rats at 20 months, but is detected in neurons with advancing age (34 months [56]). Thus, in primary neurons, cathepsin E activity is also unlikely to be the protease generating cpA.

It is formally possible that the aspartyl protease activity involved in cpA production is an autoproteolytic activity of huntingtin. Aspartyl proteases use two aspartates within their active sites and presenilin-1 has been shown to undergo endoproteolysis that requires two of its aspartates [57]. Huntingtin contains 6 aspartates (D100, D139, D140, D144, D150, and D162) within the first 171 amino acids that could form an active site. Self-digestion assays with purified MBP-tagged huntingtin failed to generate cpA (our unpublished observations) arguing against an autocatalytic property.

Our data suggest that the aspartyl protease responsible for cpA may be cell type specific. We found cathepsin D/cathepsin E like aspartyl proteases more effective in clonal striatal cells and Hela cells and gamma secretase more effective in neurons. It is likely that cathepsin D and gamma secretase are present in all cell types. It is unclear what accounts for this variation. Cell type specific differences in intracellular targeting of the expressed htt and access to available protease may be involved. Expression levels of htt may be much higher with transient transfection into fewer proliferating cells than with lentivirus-mediated delivery into neurons where exogenous protein is present at lower levels in most neurons. High levels of expressed huntingtin fragment induce autophagy and increase the activity of cathepsin D in clonal striatal cells [58]. We suggest that proteases identified to cleave htt in cell lines need to be validated by analysis of neurons.

The primary sites of cleavage for generating cpA or cpB have not been determined despite deletion analysis and alanine scanning efforts. Deletion of huntingtin 104-114 [23] and 108-113 [24] blocked cpA production in cells however it is still not clear whether this is the actual site of cleavage or a so-called "docking site" for the enzyme. Compound P1-N031 is an aspartyl protease transition state mimetic and as such might be useful to predict the primary site of cleavage within huntingtin. Unfortunately, results from a comparison of P1-N031 to structural predictions for the polypeptide region in huntingtin 101-115 did not reveal the primary site of cleavage. We and others have tried unsuccessfully to obtain enough purified product to determine the cleavage site by mass spectrometry. Using western blot analysis and epitope mapping, the smallest detectible fragment generated from full-length huntingtin in PC12 cells or from knock-in mice or was the size of exon 1 (aa1-89) [59,60]. Interestingly, this would place the site of cleavage at a group of charged polar amino acids immediately adjacent to a membrane-binding region [43-45] consistent with the hypothesis that huntingtin may slip into the catalytic pocket of presenilin if it is exposed to the aqueous environment [49].

Our studies did not reveal a protease involved in forming cpB. Although inhibitors from five protease families, including aspartyl proteases were effective in reducing cpB, none were effective in follow-up dose response analysis. We also found no compounds in our screen that inhibited cpB but not cpA. If cpA and cpB are generated from the same enzyme, it could be argued that cpB is required for cpA formation. In this scenario, inhibition of both cleavages to the same degree would result in no effective change in cpB levels, since reduced formation of cpB would be balanced by reduced breakdown of cpB to cpA. Alternatively, our assay design may not be optimal for detecting inhibition of cpB since much more cpB is produced from huntingtin 1-287 compared to cpA. Finally, compounds most effective for cpB inhibition may not have optimal permeability characteristics to access intracellular compartments where cleavage may occur.

## Conclusion

We have shown that small N-terminal huntingtin fragments known as cpA and cpB are generated in neurons in brain and primary neuron cultures and do not require additional inhibition of the proteasome to accumulate. A cell-based screen of protease inhibitors showed that the enzyme producing cpA is an aspartyl protease and in primary neurons it is likely to be gamma-secretase or a highly related enzyme. CpA formed in primary neurons using viral delivery of huntingtin aa 1-171. It is noteworthy that a cleavage product the size of cpA is detected in brain of transgenic mice expressing mutant huntingtin aa1-171 with polyglutamine expansion [2]. Given that some mutant huntingtin fragments may be innocuous and others harmful, it will be necessary in future studies to identify the sizes of cpA and cpB in order to determine whether expression of these fragments in the brain is toxic to neurons. Targeting the appropriate enzymes responsible for generating toxic fragments is likely to be a useful therapeutic target for HD. Unfortunately, gamma-secretase inhibitors have toxic side effects in peripheral tissues [32]. Therefore, study of the effects of modulators of gamma-secretase activity on huntingtin proteolysis may be more useful.

## Methods

### Compounds

Compounds were delivered as powders and resuspended in DMSO to a final dilution of 10 mM and stored at room temperature (RT) until further dilution. The proprietary compounds were coded blinding scientists to both structure and enzyme specificity. Gleevec (imatinib mesylate) was resuspended in 90% DMSO/10%H_2_0 to a stock concentration of 10 mM; LY-411,575 [32] and DAPT [33] were prepared as described and made soluble in the same carrier.

### Enzymatic activity assays

Enzymes were prepared as described [61] or obtained from R & D systems (Cathepsin D and Cathepsin E). Fluorescent assays to measure cleavage activity were performed as described [61].

### Cell-Based Assay

Cells, plasmids and transfections: X57 cells are an immortalized clonal striatal cell line made by fusion of E18 striatal neurons and N18TG2 neuroblastoma cells [28]. HeLa cells were obtained from American Type Culture Collection and grown using their recommendations. The X57 cells were grown at 37°C and 5% CO_2 _in Dulbecco's modified Eagle medium (DMEM) with high glucose and containing 10% fetal bovine serum (FBS), penicillin/ streptomycin and glutamine. X57 cells were transiently transfected with the pcDNA3 vector with an insertion of the cDNA encoding huntingtin aa1-287 with 18Q and a C-terminal HA tag (pcDNA3-htt287-18Q-HA) or huntingtin aa1-400 with 18Q or 100Q (pcDNA-htt400-18Q and pcDNA-htt400-100Q). For cell transfections in 10 cm diameter culture dishes, 25 μg plasmid DNA were diluted in 300 μl HBSS, supplemented with 120 μl Superfect (Qiagen), incubated for 15 minutes and diluted into 4.5 ml complete medium. 3 hours after transfection, cells were replated onto 24 well plates at 250,000 cells/well in 0.2 ml of complete medium.

### Compound treatments, cytotoxicity, and sample preparation

Compounds were pre-diluted at the desired concentration in complete medium starting from 10 mM DMSO stock solutions. 6 hours after DNA transfection and 3 hours after seeding the cells onto 24 well plates, 0.3 ml DMEM/FBS medium containing the test compounds was added to the cells. For the primary screen, the compounds were tested at a final dilution of 10 μM in 500 μl medium/well of the 24 well plates. Each plate was used to test 7 compounds in triplicates and for a vehicle (DMSO) control. 12 hours after start of the compound treatment, the cells were supplemented with a proteasome inhibitor to facilitate the detection of the proteolysis cleavage products. For this, 1.25 μl of a 2 mM epoxomicin (Chemicon) stock solution in DMSO was first diluted in batch with 8.75 μl of DMEM/FBS per well, then 10 μl were added to the wells to reach a final concentration of 5 μM epoxomicin. DMSO final concentration was 0.035%.

Cytotoxicity was assessed visually 16 hours after start of the compound treatment by observing the cells in each well under a microscope. The presence of detached or dead cells was indicative of cell toxicity. Cell lysates were prepared 18 hours after start of the compound treatment (24 hours post-transfection): culture medium was aspirated and replaced by 60 μl/well in lysis buffer (50 mM Tris pH 7.4, 250 mM NaCl, 5 mM EDTA, and 1% Nonidet NP40, with EDTA-free complete protease inhibitor (Roche)). After 10 minutes on ice with shaking, cells were scraped from the plates and transferred into a fresh tube and kept frozen at -80°C until further analysis. After thawing, the samples were centrifuged for 1 minute at 10,000 xg. The supernatants were collected, supplemented with 0.25 × volume of 4 × sample buffer (Invitrogen) with 0.1 M DTT (final) and boiled for 5 minutes. SDS-PAGE using E-PAGE-48 gels (Invitrogen) and western blot analysis were performed using Ab1 (against aa1-17 of huntingtin). Western blots were developed using SuperSignal West Pico stable Peroxide Solution (Pierce) according to manufacturer's instructions then exposed to film (Hyperfilm ECL, Amersham Biosciences). Films were scanned on a Hewlett Packard ScanJet 4C/T flat bed scanner acquired at 300 dpi using Deskscan II and Adobe Photoshop software. Densitometry of images was performed using SigmaScan software (Jandel Scientific) and statistical analysis was performed using Excel and GraphPad Prism. Exposures in which cpA and cpB could be easily detected in the control lanes were used for analysis. Triplicate results from compounds were compared to their respective controls (run on the same 24 well plate) from the same exposures.

### Cell-free endoprotease digestion assay

The assay is a self-digestion assay [24] modified from Lunkes et al, 2002 [23]. 50 μg protein from the 2000 ×g pellet obtained from cells was homogenized in buffer (20 mM Tris, 250 mM sucrose, 1 mM EDTA). The assay buffer was 50 mM sodium citrate pH 3.5. Reactions were incubated at 37°C for 60 minutes then neutralized by addition of 6.7 N NH_4_OH prior to analysis by SDS-PAGE and western blot. Compounds including pepstatin A were tested at 10 μM for their ability to inhibit the formation of small huntingtin fragments in the range of cpA or cpB.

### Construction of lentiviral vectors and AAV vectors encoding huntingtin

Lenti-PGK-htt1-171 (18Q and 100Q): The cDNA encoding 1-171 amino acids for both 18Q and 100Q huntingtin were digested with BamH I and Xho I, then sub-cloned into the corresponding sites of the pENTR1A vector (Invitrogen). The pENTR1A 18Q and 100Q huntingtin 1-171 amino acid constructs were digested with Nhe I and Xba I, while the lentiviral CSCW2-PGK vector backbone was digested with Nhe I and treated with calf alkaline phosphatase. The gel-isolated inserts were sub-cloned into the CSCW2-PGK vector. The vector also encoded an internal ribosome binding site just 3' to the huntingtin cDNA, followed by the cDNA for GFP.

Lenti-CMV-htt1-400 (18Q and 100Q): The cDNA encoding 1-400 amino acids for both 18Q and 100Q huntingtin were digested with BamH I and PshA I, while the pENTR1A vector (Invitrogen) was digested with BamH I and Not I. Prior to ligation with insert, the Not I site of the pENTR1A vector was blunt-ended with Mung Bean Nuclease (New England Biolabs). The pENTR1A 18Q and 100Q huntingtin 1-400 amino acid constructs were digested with BamH I and EcoR V, while the CSCW2-hNoggin-IG vector (generous gift of Miguel Esteves) was digested with Nhe I and treated with calf alkaline phosphatase. Prior to ligation, the BamH I site of the 1-400 amino acid inserts were blunt-ended with Mung Bean Nuclease. The gel-isolated inserts were sub-cloned into the CSCW2 vector. The vector also encoded an internal ribosome binding site just 3' to the huntingtin cDNA, followed by the cDNA for GFP. AAV-htt1-400 (18Q and 100Q): The construction of the AAV vector driven by CBA promoter has been previously described [11].

### Lentiviral production

Lentiviral vectors were transfected into 293T cells using FuGene 6 Reagent (Roche). Virus supernatant was isolated by centrifugation at 24,000rpm for 90 minutes at 4°C. Lentiviral pellets were resuspended in PBS/0.5% BSA solution. Virus titer was determined using RETROtek HIV-1 p24 Antigen ELISA (ZMC).

### AAV delivery into mouse striatum

The animal protocol in this study was approved by the University of Massachusetts Medical School (A-978). Mice (C57B6) received microinjections by stereotactic placement into the right striata of AAV-htt-400-18Q (3 μl per striata, N = 4 mice) or AAV-htt-100Q unilaterally (3 μl per striata, N = 4 mice) using methods previously described [11]. Mice were killed 3 days after injection and the injected striata were harvested for biochemical analysis.

### Primary neuron cultur*e*

Timed-pregnant Sprague-Dawley rats (Charles Rivers Laboratories) were sacrificed by CO_2 _inhalation and embryos (embryonic day 19) were collected in a Petri dish and placed on ice. Dissections were performed under a stereomicroscope in ice cold dissection medium (1× HBSS) containing 1 mM pyruvate, 0.6% D-glucose, 10 mM HEPES and 1% Pen-Strep. Cerebral cortices or striata were isolated, cut into pieces using forceps, and collected in a 15 ml Falcon tube. The tissue was homogenized by repeated pipetting with a fire-polished Pasteur pipette in dissection medium. Cells were centrifuged at 4°C for 5 minutes at 1000 ×g and resuspended in Neurobasal medium containing 2% B27, 1% Pen-Strep and 2 mM L-Glutamine. 300,000 cells were plated per well of 12-well plates coated with 50 μg/ml Poly-D-lysine. On day *in vitro *(DIV) 1, the cultures were infected with lentiviral vectors. Half of the medium was replaced with fresh Neurobasal medium supplemented with 2% B27, 1% Pen-Strep and 2 mM L-Glutamine on DIV4 for cultures maintained for several days. Primary rat neurons were infected DIV 2 with Lenti-PGK-htt1-171-18Q or 100Q lentivirus at 80 multiplicity of infection (moi) or at indicated moi then cultured for up to 2 weeks.

Pregnant mice (C57BL/6 strain background) were obtained from Charles River Laboratories. The animal protocol was reviewed and approved by the MGH Subcommittee on Research Animal Care (SRAC)-OLAW Assurance # A3596-01. The protocol was submitted and reviewed conforms to the USD Animal Welfare Act, PHS Policy on Humane Care and Use of Laboratory Animals, the "ILAR Guide for the Care and Use of Laboratory Animals" and other applicable laws and regulations. HD mice were of the same strain but had human exon1 with an expanded polyQ (140Q) introduced by homologous recombination into the endogenous mouse allele [14]. Homozygous Q140/Q140 mice were used to prepare cortical neuron cultures for the toxicity assay.

Primary cortical neurons were obtained from brains of embryonic mice (E15-E17) as described before [36]. Briefly, brains were dissected from embryos and after meninges were removed, cortices were incubated at 37°C for 15 min with 0.625% trypsin in Neurobasal media (NBM, supplemented with B27 and N2, 500 μg/ml streptomycin, 100 IU/ml of penicillin and 2 mM L-glutamine, all from Invitrogen, Carlsbad, CA). Trypsin was removed and Dulbecco's Modified Eagle Medium plus nutrient mix F12 (DMEM/F12, from Invitrogen) supplemented with 10% fetal bovine serum (FBS from Invitrogen), N2 supplement and penicillin/streptomycin was added. Cortices were treated with 12.5 μg/ml of DNAse I (Sigma, St. Louis, MO) in DMEM/F12 for 5 min at 37°C and homogenized. Cell suspension was then diluted in NBM supplemented with 15 μM L-glutamate and 10 μM 2-mercaptoethanol (Invitrogen). Neurons were plated at 800,000 cells/ml in culture dishes pre-coated with poly-L-lysine 0.2 mg/ml in borate buffer pH 8.0 (30-70 kDa from Sigma) for 24 h followed for 1 h coating period with 10% FBS (DMEM/F12 plus 10% FBS). Cytosine β-D-arabinofuranoside (AraC) was added at second day *in vitro *(DIV) for 24 h, and then media was completely replaced with fresh NBM. Half of the volume of medium was replaced every third day. A quantitative assessment using markers for neural cell types including Beta III-tubulin for neurons, glial fibrillary acidic protein for astrocytes and cd68 for microglia showed that the majority of cells are neurons (99.5%). Primary mouse neurons were infected DIV 1 with Lenti-PGK-htt1-171-18Q or 100Q lentivirus at 160 moi then cultured for up to 2 weeks.

For compound treatment experiments, primary mouse neurons were infected DIV 1 with Lenti-PGK-htt1-171-18Q lentivirus at 160 moi. Ara C treatment was omitted. After 8 hours, the cells were then treated with compounds re-suspended in Neurobasal media to appropriate concentrations and cultured for an additional 16 hours (24 hours for infection, 16 hours for treatment). Cells were harvested 24 hours post-infection in NP40 lysis buffer + protease inhibitor tablet. 12 μl of lysate was loaded onto 4-12% Bis-Tris gels and analyzed by western blot and densitometry analysis as described above. Membranes were probed with Ab1 (0.5 μg/ml) and Beta III-Tubulin (1:1000; Sigma).

### SiRNA experiments

siRNA targeting cathepsin D (Dharmefect SmartPool) and a control siRNA that does not target any known sequence in the mouse genome (non-target) were obtained from Dharmacon. SiRNAs were introduced to primary neurons using Dharmafect reagent 1 (Thermo, Inc). At DIV5, neurons were incubated for 1 hour with antibiotic free Neurobasal medium before transfection to acclimate cells to experimental conditions. 2.8 mL of complete antibiotic free medium was added to complexed Dharmafect/siRNA solution (siRNA final concentration 20 nM) for a total volume of 3.5 mL and incubated for 48 hours. Then neurons were harvested in 75-100 uL of lysis buffer (50 mM Tris, 250 mM NaCl, 5 mM EDTA, 1% NP40, pH 7.4) with mini-EDTA-free cocktail protease inhibitor tablet (Roche) and analyzed by western blot using anti-cathepsin D antibody (Oncogene Scienece).

### Immunoprecipitation

HD neurons were lysed in IP buffer (50 mM Tris pH 7.4, 250 mM NaCl, 1% NP40, 5 mM EDTA + protease inhibitor tablet (Roche)). Insoluble material removed by centrifugation at 14,000 ×g , protein levels of supernatants were determined by Bradford assay, and 1 mg per protein condition was incubated with protein G sepharose beads (GE Healthcare) plus monoclonal anti-htt1-17 (mAb2B7, [39]) overnight at 4°C. Sepharose beads were washed 3 times in IP buffer then 1 time in PBS, then bound protein was eluted by boiling in 75 μl sample buffer (Invitrogen) + DTT and analyzed by western blot using polyclonal anti-htt1-17 (Ab1). No primary antibody and buffer alone were run as controls.

### Western blot analysis of brain lysates, primary neurons and clonal striatal cell

Brains were removed 3 days after injection with AAV-htt1-400-18Q or AAV-htt1-400-100Q and rapidly frozen. The area around the injection site was dissected on ice and homogenized in 10 volumes lysis buffer + protease inhibitor. After incubating 10 minutes at RT, lysates were centrifuged at 10,000 ×g for 2 minutes and the protein concentration of the supernatant was determined using the Bradford assay. 20 μg of protein was separated by SDS-PAGE on 4-12% Bis-Tris, 4-20% Tris-glycine or 3-8% Tris-acetate gels (Invitrogen) and analyzed by western blot using anti-huntingtin antibody Ab1 (made against huntingtin aa1-17) at 0.5 μg/mL, anti-cathepsin D antibody (Oncogene Science ) at 2.5 μg/ml, anti-cathepsin E antibody (R&D Systems) at 0.2 μg/ml or anti-βIII tubulin (Sigma) at 1:1000.

### Gamma-Secretase Assay

Huntingtin 1-171-18Q with an N-terminal FLAG-tag (Fhtt171-18Q) was translated *in vitro *(TNT T7 Quick Coupled Transcription/Translation System, Promega) then immunopurified with M2-speharose (Sigma) and eluted with TBS pH 7.1 as described previously [43]. The CHAPSO-soluble membrane fraction was prepared according to Li et al. [35] from SY5Y cells which are reported to contain all four components of gamma-secretase complex [34]. In vitro digestions contained equal volumes of purified huntingtin 1-171-18Q and 50 mM PIPES pH6.8, 5 mM MgCl_2_, 5 mM CaCl_2_, 150 mM KCl, 0.25% CHAPSO buffer. The CHAPSO-soluble membrane fraction was added at 125 μg/ml with and without 1 μM DAPT. Reactions were incubated for 2 hours or 5 hours at 37°C then stopped with SDS-PAGE sample buffer and analyzed by SDS-PAGE and western blot.

## Abbreviations

AAV: adeno-associated virus; cpA: cleavage product A; cpB: cleavage product B; HD: Huntington disease; htt: huntingtin.

## Competing interests

The authors declare that they have no competing interests.

## Authors' contributions

KBK designed and oversaw the study and prepared the manuscript, ES assisted with the assays, western blots and analysis, JA performed cell culture, assays, western blots and analysis, PR performed densitometry, DB participated in compound assays, LS assisted in setting up initial experiments, NM made the lentivirus, AV and HJ prepared primary neuronal cultures, DK assisted in design and manuscript preparation, JP and DC provided compounds, CB selected and provided compounds, ME and NA made the AAV constructs, PP and RK provided compounds and assisted in experimental design, and MD aided in design of the study and manuscript preparation. All authors have read and approved the manuscript.

## Supplementary Material

Additional file 1 **Figures S1-S5**. Results from the initial screen in clonal striatal X57 cells for small compounds that reduce levels of cpA or cpB are shown in graphical form in Figure S1. In Figure S2, select compounds were tested using an in vitro self-digestion assay of wild-type and mutant huntingtin fragments. Figure S3 shows protein levels of cathepsin D & E in cells and in brain. Figure S4 shows cathepsin D protein levels after mRNA silencing and a lack of effect on cpA production in lentivirus infected primary neurons. Finally, Figure S5 shows that treatment with the gamma-secretase inhibitor DAPT reduced levels of small huntingtin fragments in Q140/Q140 knock-in mice, correlating with increased survival. Click here for file

Additional file 2 **Table S1. Compounds that reached < 50% and/or p < 0.05 for cpA and cpB in clonal striatal cells**. This table summarizes all positive hits from the initial screen in clonal striatal X57 cells expressing exogenous huntingtin 1-287-18Q for small compounds that reduce levels of cpA or cpB. The data for all compounds tested is shown in graphic form in Additional file 2. Click here for file
