# Supplementary material for: Huntingtin cleavage product A forms in neurons and is reduced by gamma-secretase inhibitors
Source: Mol Neurodegener. 2010 Dec 14;5:58. doi: 10.1186/1750-1326-5-58 (PMC3018386; doi:10.1186/1750-1326-5-58)
Supplement: Additional file 1 — Figures S1-S5. Results from the initial screen in clonal striatal X57 cells for small compounds that reduce levels of cpA or cpB are shown in graphical form in Figure S1. In Figure S2, select compounds were tested using an in vitro self-digestion assay of wild-type and mutant huntingtin fragments. Figure S3 shows protein levels of cathepsin D & E in cells and in brain. Figure S4 shows cathepsin D protein levels after mRNA silencing and a lack of effect on cpA production in lentivirus infected primary neurons. Finally, Figure S5 shows that treatment with the gamma-secretase inhibitor DAPT reduced levels of small huntingtin fragments in Q140/Q140 knock-in mice, correlating with increased survival. [file 1750-1326-5-58-S1.PDF]

Additional file 1

Title: Figures S1-S5.

Description: Results from the initial screen in clonal striatal X57 cells for small compounds that reduce levels of cpA or cpB are shown in graphical form in Figure S1. In Figure S2, select compounds were tested using an in vitro self-digestion assay of wild-type and mutant huntingtin fragments. Figure S3 shows protein levels of cathepsin D & E in cells and in brain. Figure S4 shows cathepsin D protein levels after mRNA silencing and a lack of effect on cpA production in lentivirus infected primary neurons. Finally, Figure S5 shows that treatment with the gamma-secretase inhibitor DAPT reduced levels of small huntingtin fragments in Q140/Q140 knock-in mice, correlating with increased survival.

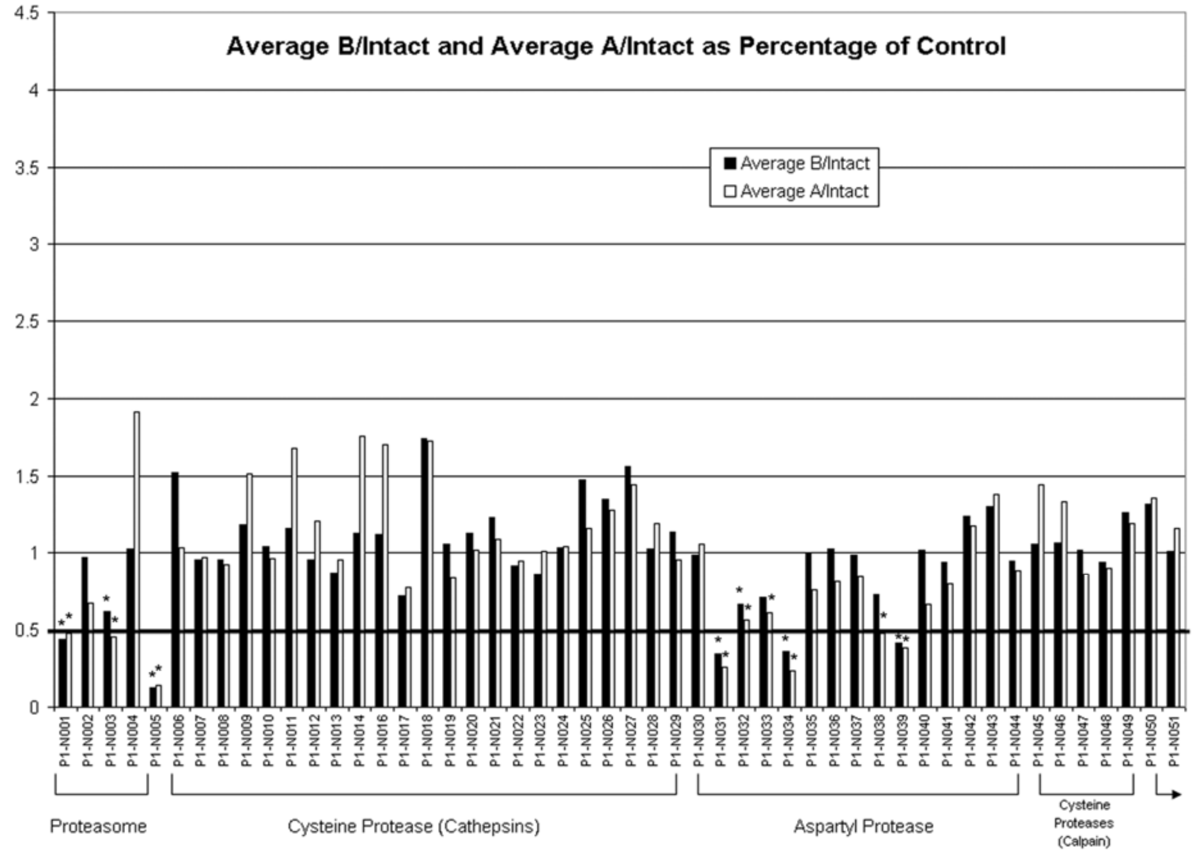
Average B/Intact and Average A/Intact as Percentage of Control
Average B/Intact
Average A/Intact
4.5
4
3.5
3
2.5
2
1.5
1
0.5
0
P1-N001
P1-N002
P1-N003
P1-N004
P1-N005
P1-N006
P1-N007
P1-N008
P1-N009
P1-N010
P1-N011
P1-N012
P1-N013
P1-N014
P1-N016
P1-N017
P1-N018
P1-N019
P1-N020
P1-N021
P1-N022
P1-N023
P1-N024
P1-N025
P1-N026
P1-N027
P1-N028
P1-N029
P1-N030
P1-N031
P1-N032
P1-N033
P1-N034
P1-N035
P1-N036
P1-N037
P1-N038
P1-N039
P1-N040
P1-N041
P1-N042
P1-N043
P1-N044
P1-N045
P1-N046
P1-N047
P1-N048
P1-N049
P1-N050
P1-N051
Proteasome
Cysteine Protease (Cathepsins)
Aspartyl Protease
Cysteine Proteases (Calpain)

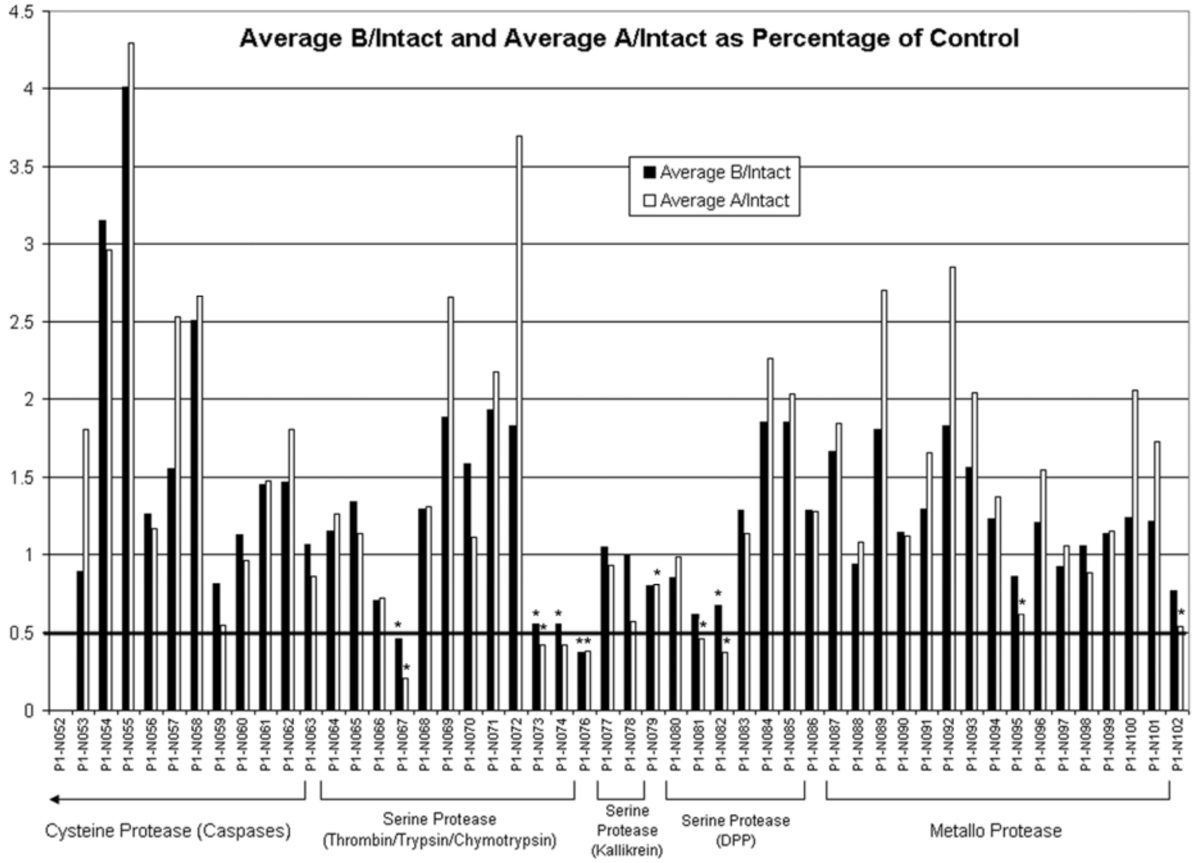
Average B/Intact and Average A/Intact as Percentage of Control
Average B/Intact
Average A/Intact
4.5
4
3.5
3
2.5
2
1.5
1
0.5
0
P1-N052
P1-N053
P1-N054
P1-N055
P1-N056
P1-N057
P1-N058
P1-N059
P1-N060
P1-N061
P1-N062
P1-N063
P1-N064
P1-N065
P1-N066
P1-N067
P1-N068
P1-N069
P1-N070
P1-N071
P1-N072
P1-N073
P1-N074
P1-N076
P1-N077
P1-N078
P1-N079
P1-N080
P1-N081
P1-N082
P1-N083
P1-N084
P1-N085
P1-N086
P1-N087
P1-N088
P1-N089
P1-N090
P1-N091
P1-N092
P1-N093
P1-N094
P1-N095
P1-N096
P1-N097
P1-N098
P1-N099
P1-N100
P1-N101
P1-N102
Cysteine Protease (Caspases)
Serine Protease (Thrombin/Trypsin/Chymotrypsin)
Serine Protease (Kallikrein)
Serine Protease (DPP)
Metallo Protease

Figure S1 - (Graphs 1& 2) Screening results of a focused library of 102 proprietary compounds known to inhibit specific proteases using a cell-based assay to find compounds that inhibit cpA and/or cpB production. The Y-axis on graphs is the ratio of signal for the protease product to signal for intact exogenously expressed protein. Bars represent the mean of 3 replicates. * indicates statistical significance compared to samples treated with DMSO ($p<0.05$, student t-test, n=3). Triplicate wells of clonal striatal cells transiently expressing H287-18Q-HA were treated with compounds at 10 μM final concentration for 21 h. Epoxomicin (5 μM) was added for the final 6 h and cells were harvested 24 hours post-transfection. Lysates were analyzed by western blots probed with anti-huntingtin antiserum Ab1 (0.5 μg/ml).

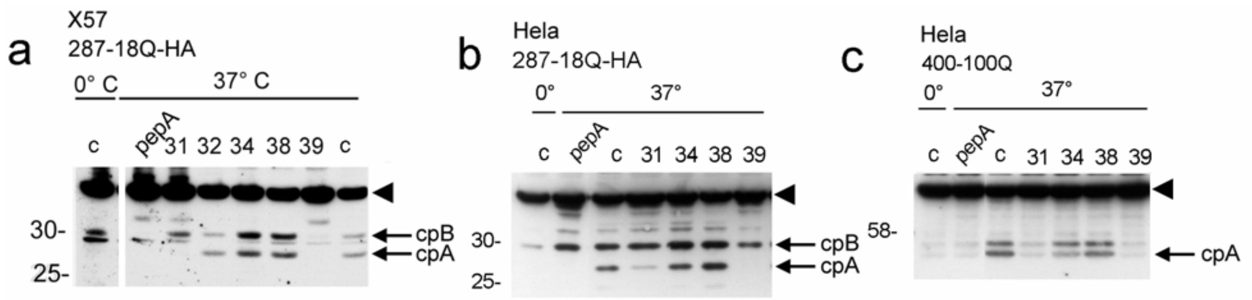

Figure S2 - Small molecules inhibit formation of cpA from wild-type and mutant huntingtin fragments *in vitro*. (**a**) Clonal striatal (X57) cell-lysates with H287-18Q-HA, (**b**) HeLa cell-lysates with H287-18Q-HA, (**c**) HeLa cell-lysates with H400-100Q. Self-digestion assays using the P1 fraction from clonal striatal cells (X57 cells) or HeLa cells were performed as described (see methods) and analyzed by SDS-PAGE and western blots using anti-huntingtin antiserum Ab1. Compounds were added to reactions at a final concentration of 10 μM (P1-0XX code with XX indicated across top of blots). Pepstatin A (Pep A) was used as a positive control. Reactions with no compound 'c' incubated at 0^oC produce very little cpA (left side of each blot). Reactions incubated at 37^oC in the absence of compound "c" produce cpA from H287-18Q **(a, b**) and from H400-100Q (**c**). Arrowhead indicates intact expressed protein; arrows indicate cleavage products. Note that for 287-18Q-HA an increase in the intact form occurs in the presence of pepstatin A or by compound P1-N031 or P1-N039.

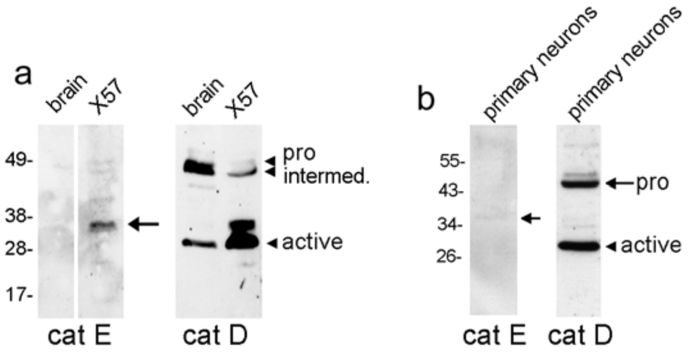

Figure S3 - Cathepsin D and cathepsin E levels in cells and in mouse brain. (a) Western blots of lysates from total mouse brain and clonal striatal cells (X57) run in parallel and probed with anti-cathepsin E or anti-cathepsin D antibodies. Molecular weight in kDa is indicated to the left. In left blot, cathepsin E migrates at ~38kDa (arrow) and is only present in X57 cells. In right blot, pro-cathepsin D and the partially processed intermediate form of cathepsin D (52 and 46 kDa) are indicated with arrowheads; the processed, active form of cathepsin D (32kDa) is indicated with an arrowhead. The band migrating slightly higher than the active cathepsin D may be cross reactivity of the anti-cathepsin D antibody with cathepsin E. **(b)** Cathepsin D is present in primary mouse neurons. Pro-cathepsinD is indicated with arrow; the processed, active form of cathepsin D is indicated with an arrowhead. Cathepsin E detected at very low levels in primary neurons (small arrow).

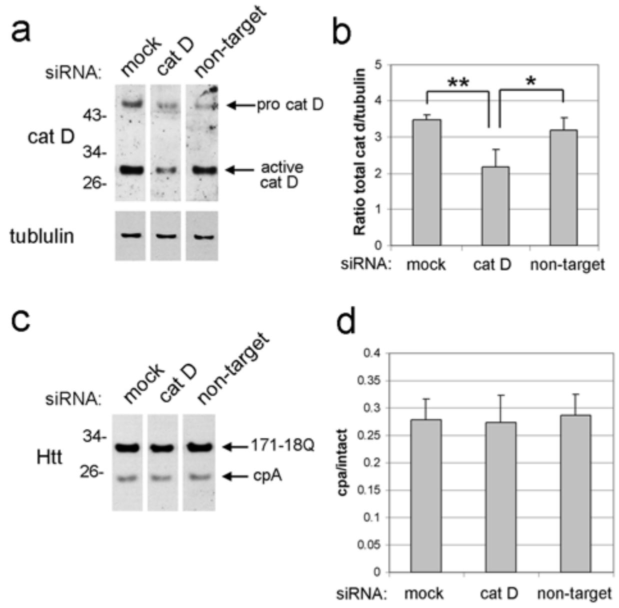

Figure S4 - Effects of cathepsin D mRNA silencing on cpA production in primary neurons. (a and b). Levels of cathepsin D after siRNA knockdown in primary neurons also infected with lentivirus 171-18Q. Western blots show pro and active forms of cathepsin D (arrows). Beta-3-tubulin was used as a loading control. In b, densitometry levels of total cathepsin D (pro + active) were standardized to tubulin. * $p<0.05$, ** $p<0.01$, n=3 unpaired t-test. **(c and d)** Levels of cpA with cathepsin D knockdown. Shown are blots using the same lysates as in a and b. Blots probed with anti-huntingtin antibody Ab1. In c, arrows indicate expressed intact 171-18Q and cpA. In d, densitometry results report cpA levels as a ratio of cA to intact expressed htt171-18Q. As controls, we used a non-targeting siRNA or siRNA transfection reagents alone. For all conditions, cells were infected after 24 hours with Lenti-htt1-171-18Q. Cells were harvested after an additional 24 hours and analyzed by SDS-PAGE and western blot.

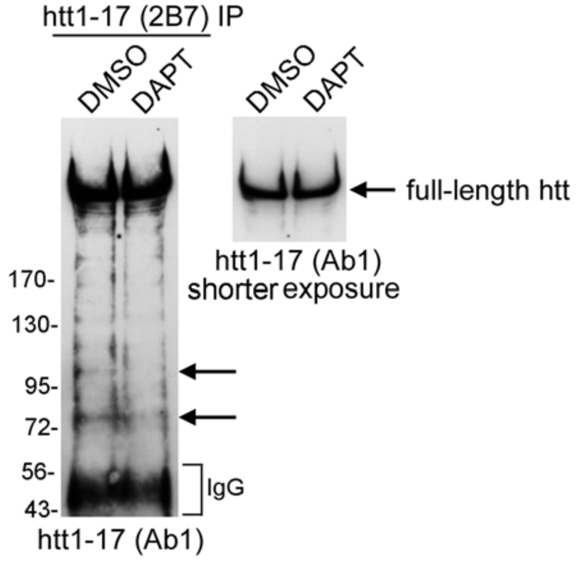

Figure S5 – An inhibitor of gamma-secretase reduces N-terminal huntingtin fragments in cortical HD neurons homozygous for full-length mutant huntingtin (Q140/Q140). HD cortical neurons were treated continuously with DMSO or 0.1 μM DAPT starting at 3 DIV. At 10 DIV, neurons were lysed and mutant huntingtin was immunoprecipitated using a monoclonal antibody against htt1-17 (mAb 2B7) and probed with polyclonal anti-htt1-17 (Ab1). Arrows indicate full-length mutant huntingtin and N-terminal mutant huntingtin fragments reduced by DAPT treatment at ~80 kDa and ~100 kDa. Small blot on right shows a shorter exposure of immunoprecipitated full-length mutant huntingtin. The 72 kDa fragment detected at 23 DIV was not observed at 10 DIV even at long exposures.
