# Supplementary material for: Huntingtin cleavage product A forms in neurons and is reduced by gamma-secretase inhibitors
Source: Mol Neurodegener. 2010 Dec 14;5:58. doi: 10.1186/1750-1326-5-58 (PMC3018386; doi:10.1186/1750-1326-5-58)
Supplement: Additional file 2 — Table S1. Compounds that reached < 50% and/or p < 0.05 for cpA and cpB in clonal striatal cells. This table summarizes all positive hits from the initial screen in clonal striatal X57 cells expressing exogenous huntingtin 1-287-18Q for small compounds that reduce levels of cpA or cpB. The data for all compounds tested is shown in graphic form in Additional file 2. [file 1750-1326-5-58-S2.PDF]

Additional file 2

Title: Table S1. Compounds that reached <50% and/or p<0.05 for cpA and cpB in clonal striatal cells.

Description: This table summarizes all positive hits from the initial screen in clonal striatal X57 cells expressing exogenous huntingtin 1-287-18Q for small compounds that reduce levels of cpA or cpB. The data for all compounds tested is shown in graphic form in Additional file 2.

Table S1. Compounds that reached <50% and/or p<0.05 for cpA and cpB

Compound	Target	% of control for cpB, t test	% of control for cpA, t test
**P1-N001**	Proteasome	44.3%, p<0.05	48.1%, p<0.05
**P1-N003**	Proteasome	61.9%, p<0.05	45.8%, p<0.05
**P1-N005**	Proteasome	12.8%, p<0.05	14%, p<0.05
**P1-N031**	Aspartyl protease	34.4%, p<0.05	26%, p<0.05
**P1-N032**	Aspartyl protease	66.7%, p<0.05	
**P1-N034**	Aspartyl protease	35.8%, p<0.05	23.3%, p<0.05
**P1-N038**	Aspartyl protease		47.9%, p<0.05
**P1-N039**	Aspartyl protease	41.5%, p<0.05	38.4%, p<0.05
**P1-N067**	Serine proteases (thrombin/trypsin/chymotrysin)	45.9%, p<0.05	20.6%, p<0.05
**P1-N073**	Serine proteases (thrombin/trypsin/chymotrysin)	55.4%, p<0.05	41.4%, p<0.05
**P1-N074**	Serine proteases (thrombin/trypsin/chymotrysin)	55%, p<0.05	41.4%
**P1-N076**	Serine proteases (thrombin/trypsin/chymotrysin)	37.3%, p<0.05	37.6%, p<0.05
**P1-N079**	Serine proteases (kallikrein)		80.4%, p<0.05
**P1-N081**	Serine proteases (DPP)	61.4%, p<0.05	45.5%, p<0.05
**P1-N082**	Serine proteases (DPP)	66.6%, p<0.05	36.9%,
**P1-N095**	Metallo protease		61.6%, p<0.05
**P1-N102**	Metallo protease		53.4%, p<0.05
